# Immune checkpoint inhibitor-induced myocarditis with atrioventricular conduction abnormalities: a case report

**DOI:** 10.1016/j.esmogo.2026.100320

**Published:** 2026-04-14

**Authors:** A. Strijdhorst, T.S.J. Opstal, L.F.H.J. Robbers, M. Labots, H.W.M. van Laarhoven

**Affiliations:** 1 Department of Medical Oncology, Amsterdam University Medical Center, Vrije Universiteit, Amsterdam, The Netherlands; 2 Cancer Center Amsterdam, Cancer Treatment and Quality of Life, Amsterdam, The Netherlands; 3 Amsterdam Cardiovascular Sciences, Atherosclerosis & Ischemic Syndromes, Amsterdam, The Netherlands; 4 Department of Cardiology, Amsterdam University Medical Center, University of Amsterdam, Amsterdam, The Netherlands; 5 Department of Medical Oncology, Amsterdam University Medical Center, University of Amsterdam, Amsterdam, The Netherlands

**Keywords:** immune checkpoint inhibitors, nivolumab, myocarditis, cardio-oncology, atrioventricular conduction abnormalities

## Abstract

Immune checkpoint inhibitors (ICIs) are increasingly used in the treatment of several cancer types, but can cause immune-related adverse events (irAEs) such as myocarditis. ICI-induced myocarditis is rare but potentially life threatening and may present with subtle or atypical symptoms, including conduction abnormalities. We describe a case of nivolumab-induced myocarditis in a patient receiving adjuvant therapy for esophageal cancer, presenting with high-grade atrioventricular block and neuromuscular symptoms, highlighting the diagnostic and therapeutic challenges of ICI-induced myocarditis. The case underscores the importance of early recognition, comprehensive diagnostic evaluation, prompt initiation of immunosuppressive therapy, and individualized management of conduction disturbances. Early detection and intervention are key to mitigating risks and improving outcomes in this rare but serious irAE.

## Highlights

• ICI-induced myocarditis is rare but potentially life threatening.
• Symptoms may be subtle or atypical, such as isolated conduction abnormalities.
• Early diagnosis and immunosuppressive therapy are crucial due to high mortality.
• High-grade AV block can be reversible with systemic immunosuppressive treatment.
• Supportive management, such as pacing, should be considered on an individual basis.

## Introduction

Immune checkpoint inhibitors (ICIs) have revolutionized the treatment landscape for various malignancies, demonstrating significant improvements in survival across neoadjuvant, adjuvant, and metastatic settings.^1^ However, a significant proportion of patients develop immune-related adverse events (irAEs), which can affect any organ system. Among these, myocarditis is a rare but potentially life-threatening irAE, characterized by variable clinical presentation and disease severity.^2^^,^^3^ Early recognition and management are crucial, given the risk of severe cardiac complications. Here, we present a case of a patient with esophageal cancer who developed conduction abnormalities following a single cycle of adjuvant nivolumab, highlighting the diagnostic and therapeutic challenges associated with immune-related (IR) myocarditis.

## Case presentation

**Figure 1:**
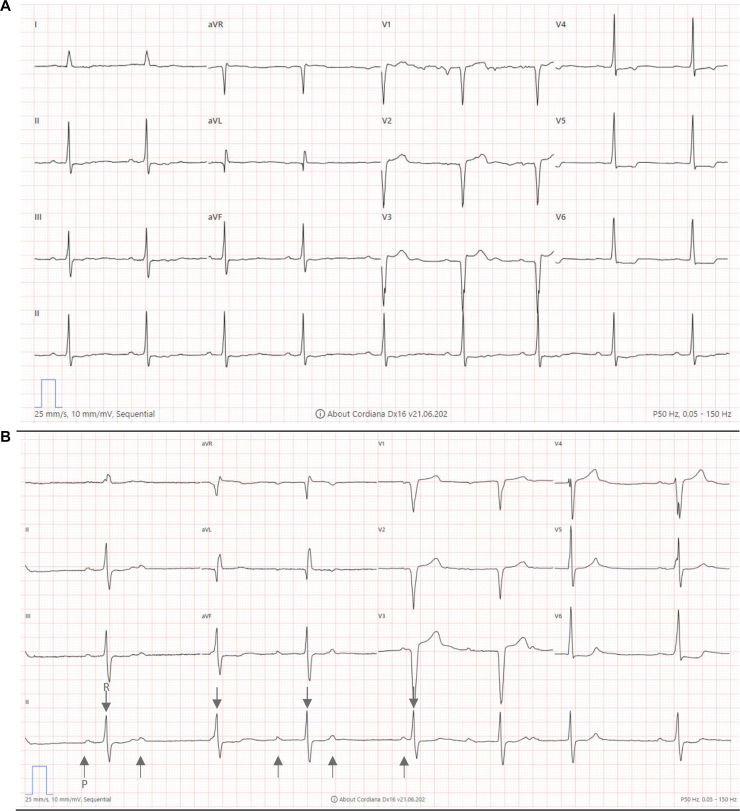
Electrocardiographic (ECG) changes before and after surgery. (A) ECG before surgery showing a first-degree atrioventricular (AV) conduction delay (PQ 226 ms). (B) ECG at presentation showing sinus bradycardia (48 bpm) with a new third-degree AV block and an escape rhythm with left bundle branch block-like configuration. Arrows indicate dissociation between atrial (P) and ventricular (R) rate.

A 64-year-old male presented with ptosis and exertional dyspnea 4 weeks after administration of the first cycle of adjuvant nivolumab, which followed treatment of stage III esophageal cancer. His medical history included sarcoidosis, in remission since 2002. In March 2023, he was diagnosed with cT3N1M0 esophageal adenocarcinoma and underwent neoadjuvant chemoradiotherapy (paclitaxel 50 mg/m^2^ plus carboplatin AUC2 weekly for 5 weeks, concomitant with radiation, 41.4 Gy, given in 23 fractions of 1.8 Gy), followed by esophagectomy with a cervical anastomosis. Post-operative complications had included anastomotic leakage, leaving persistent anastomotic stenosis requiring weekly dilations. Given the incomplete pathologically documented response on neoadjuvant treatment and based on improved disease-free survival demonstrated in the Checkmate 577 trial, adjuvant nivolumab was initiated 14 weeks after surgery in November 2023 (480 mg every 4 weeks for up to 1 year).^4^ At baseline, his medication included esomeprazole 40 mg daily. On physical examination, he had an Eastern Cooperative Oncology Group performance status of 1 (i.e. restricted in physically strenuous activity but ambulatory), with no observed abnormalities. Laboratory tests revealed normal C-reactive protein (3.0 mg/l), mild anemia (Hb 7.5 g/dl), elevated eosinophils (1.56 × 10^5^), decreased lymphocytes (0.65 × 10^5^), and normal renal and hepatic function. Before surgery, electrocardiogram (ECG) showed a first-degree atrioventricular (AV) conduction delay (PQ 226 ms), a minor aspecific intraventricular conduction delay (QRS 106 ms), Qs in V1-3 and mild ST elevations V1-2 with ST depressions inferolateral, while an echocardiogram showed no significant abnormalities (Figure 1A).

Four weeks after the first nivolumab administration, routine blood tests showed elevated transaminases [aspartate aminotransferase (AST) 168 U/l, alanine aminotransferase (ALT) 138 U/l, and lactate dehydrogenase (LDH) 601 U/l)] with normal bilirubin levels, at that time interpreted as grade 2 IR hepatitis. After the second cycle was postponed for 1 week, transaminases had increased within grade 2 margins (AST 225 U/l, ALT 205 U/l, LDH 802 U/l) and the patient developed new symptoms of mild left-eye ptosis and mild exertional dyspnea. He was admitted to the emergency department for a suspected irAE, such as ICI-induced (myocarditis with) myositis and/or myasthenia gravis overlap syndrome.

### Initial workup

Upon admission, the patient reported ptosis and exertional dyspnea, but denied chest pain, dizziness, diplopia, muscle weakness, pain, or swallowing difficulties. Physical examination revealed normal vital signs (heart rate 59 bpm, blood pressure 133/57 mmHg, oxygen saturation 98%, and respiratory rate 12 per min), bilateral ptosis improving with an ice test, and mild hoarseness. No other neurological, cardiac, or systemic abnormalities were identified. ECG demonstrated sinus bradycardia (48 bpm) with a new third-degree AV block and an escape rhythm with left bundle branch block-like configuration (Figure 1B). Echocardiography showed normal biventricular function without pericardial effusion. Laboratory tests revealed markedly elevated creatinine kinase (CK) levels (4226 U/l), high-sensitivity troponin T (878 ng/l), and N-terminal pro-B-type natriuretic peptide (NT-proBNP) (2161 ng/l). Based on these findings, nivolumab-induced myositis with myocarditis ± myasthenia gravis was diagnosed, and the patient was admitted to the cardiac care unit for monitoring. The increased transaminases, with AST exceeding ALT, were considered to be secondary to muscle damage rather than IR hepatitis; serology had excluded a viral cause.

### Diagnosis and management

Nivolumab-induced myocarditis was diagnosed based on clinical presentation and context, markedly increased CK levels, cardiac biomarkers, and ECG abnormalities. Mild ptosis with fluctuating symptoms, confirmed by the ice test, raised the possibility of ocular myasthenia gravis. However, no myasthenia-related antibodies could be detected.

**Figure 2:**
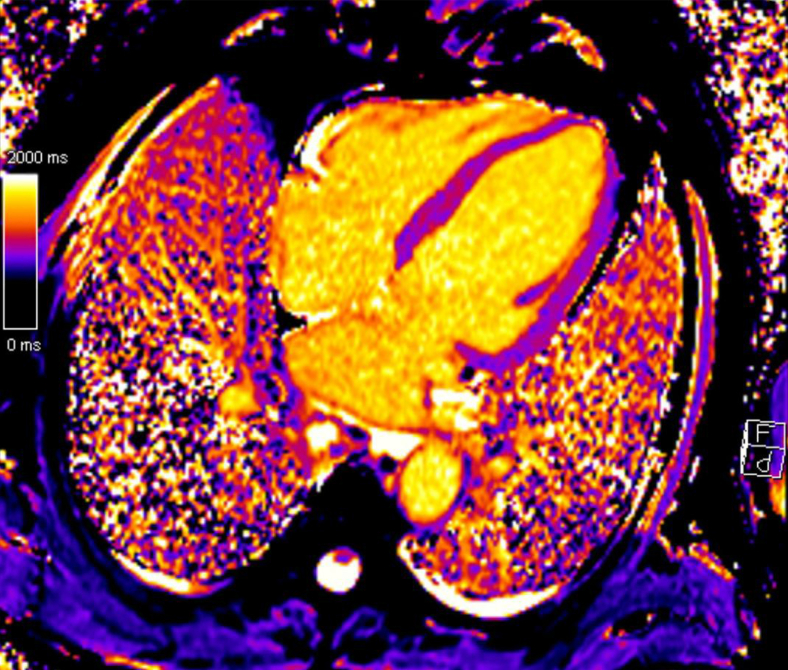
Cardiac magnetic resonance (CMR) T1 map in four-chamber view, showing an increased T1 relaxation value in the septal area, suggestive of local inflammation and/or edema.

**Figure 3:**
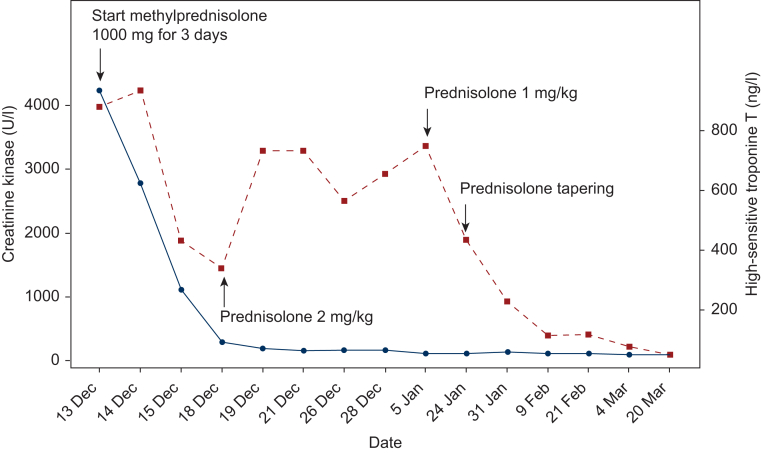
Creatine kinase levels (blue line) show a rapid decrease, whereas high-sensitive troponin T levels (red line) typically exhibit a delayed decline following initiation of immunosuppressive treatment.

Cardiac magnetic resonance imaging (MRI) showed preserved biventricular function with globally elevated myocardial T1 and T2 values, most pronounced in the septal and inferior segments, indicating diffuse myocardial inflammation/edema consistent with myocarditis. Absence of granulomas or fibrosis on late gadolinium enhancement imaging made cardiac sarcoidosis less likely (Figure 2). Following European Society for Medical Oncology (ESMO) guidelines, high-dose intravenous methylprednisolone (1000 mg/day for 3 days) was initiated, followed by oral prednisone (2 mg/kg daily) with tapering predominantly based on CK levels; troponin T levels remained >600 ng/l for 3 weeks.^5^ This approach led to resolution of the ptosis within days of methylprednisolone initiation, a rapid decrease of CK, delayed decline of high-sensitive troponin T levels (Figure 3), as is more commonly observed, and an improvement in the severity of the AV block. Despite immunosuppressive therapy, the AV block persisted. Serial ECGs and 24-h Holter monitoring confirmed second- to third-degree AV block, without symptoms. A stress ECG showed improved and consistent 1 : 1 AV conduction during exercise. For this reason, and given the absence of syncope or other high-risk features, pacemaker implantation was deferred. The patient remained hospitalized for 2.5 weeks and was discharged under close monitoring, including weekly biomarker assessment and biweekly ECG evaluation. Prednisone was tapered and discontinued over 3 months without evidence of myocarditis relapse. Upon initial admission, nivolumab treatment had been discontinued permanently.

Two months after discharge, a 24-h Holter monitor showed improved AV conduction with predominantly first-degree AV block and an occasional second-degree AV block, Mobitz type I (Wenckebach). Six months after the initial presentation with myocarditis, the patient was diagnosed with locally recurrent esophageal cancer and underwent chemoradiotherapy. Due to his limited life expectancy, he declined further cardiac imaging. He remained under outpatient follow-up by both cardiology and oncology, with no further episodes of cardiac or neurological irAEs. Unfortunately, he was later diagnosed with metastatic esophageal cancer and died 15 months later after the initial presentation.

## Discussion

This case illustrates a rare presentation of ICI-induced myocarditis, manifesting primarily as high-grade AV block, yet occurring at a typical timepoint, within 1 month after the first cycle of adjuvant nivolumab. While IR myocarditis is an uncommon (<1%) but serious complication of ICI therapy, conduction abnormalities such as second- or third-degree AV block are reported in ∼8%-17% of cases, and may be the initial or sole manifestation.^6^^,^^7^ In our patient, the AV block developed in the absence of overt heart failure or chest pain, highlighting the importance of considering myocarditis in patients with even subtle symptoms such as ptosis and exertional dyspnea. Most cases of IR myocarditis occur early during ICI therapy, with the majority of cases presenting within the first four cycles, with cases described only after 3 days of initiation.^6^, ^7^, ^8^ However, late presentations have also been described, underscoring the need for ongoing vigilance throughout treatment.

Diagnosing IR myocarditis can be challenging due to the heterogeneity of clinical presentation and frequent overlap with other irAEs, such as myositis and myasthenia gravis. Given the possible nonspecific and subtle initial symptoms, the diagnosis of IR myocarditis relies on a combination of clinical suspicion and objective findings. Current guidelines recommend a multimodal approach, integrating clinical assessment with laboratory and imaging findings.^5^^,^^9^^,^^10^ Key diagnostics include elevated cardiac biomarkers, ECG abnormalities and cardiac MRI.^5^^,^^10^ In our case, the diagnosis was based mainly on a combination of elevated cardiac biomarkers (troponin and CK) as well as progression from first-degree to third-degree AV block. ECG findings in IR myocarditis are highly heterogenous and may include ST segment elevation in various leads, sinus tachycardia, right bundle branch block, ventricular tachycardia, QT prolongation and asystole.^7^^,^^8^^,^^11^ The prognostic significance of pre-existing first-degree AV block in this context remains unclear, as current literature focuses on ECG abnormalities present at the time of diagnosis rather than baseline conduction disturbances. Cardiac MRI can be a valuable tool for confirming IR myocarditis given the high sensitivity of tissue characterization by T1 and T2 mapping, and can help exclude alternative diagnoses such as cardiac sarcoidosis, as was relevant in our patient.^5^^,^^12^

Treatment typically involves high-dose intravenous methylprednisolone, followed by a gradual taper with oral prednisone.^5^ In our patient, this approach led to rapid improvement in symptoms and biomarkers (troponin and CK), with normalization of conduction on ECG over time. For cases refractory to steroids, additional immunosuppressive agents such as abatacept (currently under investigation), mycophenolate mofetil, or tocilizumab may be considered, although evidence for optimal treatment strategy is still emerging and remains heterogeneous in the literature.^5^^,^^8^

Supportive management of conduction disturbances is guided by the patient’s clinical status. In our hemodynamically stable patient without syncope or high-risk features, pacemaker implantation was deferred. However, pacing can be indicated in IR myocarditis patients with high-grade or complete AV block who are either symptomatic, hemodynamically unstable, or have an unreliable escape rhythm.^7^^,^^13^ While persistent conduction abnormalities requiring permanent pacing are not uncommon, 30%-60% of patients experience complete or partial recovery of AV conduction following immunosuppressive therapy.^8^^,^^14^ Since the AV block in IR myocarditis may be reversible, an initial temporary pacing strategy is preferred. An active fixation right ventricular lead with an external pacemaker can be considered, potentially allowing pacing support to be maintained for several weeks.^12^ A recent prediction model has identified troponin levels, thymoma, low QRS voltage, reduced left ventricular ejection fraction, and cardiomuscular symptoms as predictors of adverse outcomes in IR myocarditis at 30 days.^15^ Although this score may guide risk stratification and treatment decisions, data on optimal management strategies and long-term outcomes for IR myocarditis with severe conduction abnormalities remain limited.

Compared with previously reported cases of ICI-induced myocarditis, which often present with symptomatic heart failure, symptomatic arrhythmias, or require pacing, our case is distinguished by isolated conduction disease, successful treatment with corticosteroids, reversibility of AV block without pacing, and ongoing improvement in both biomarkers and conduction over time.^7^^,^^8^^,^^11^^,^^14^^,^^16^^,^^17^ Given the rarity of this presentation and the absence of randomized trials, such case reports are essential for raising awareness among oncologists and cardiologists, informing clinical management, and contributing to risk stratification by highlighting the spectrum of possible presentations and outcomes in ICI-induced myocarditis.

In summary, this case highlights the importance of early recognition and prompt management of ICI-induced myocarditis, a potentially life-threatening complication that can present at any time during therapy. Unlike the more frequent presentations involving symptomatic heart failure or arrhythmias, in our patient myocarditis manifested primarily as isolated conduction abnormalities with a second- to third-degree AV block. The subtle initial complaints, such as mild ptosis and exertional dyspnea, underscore the need for clinical vigilance, as even nonspecific complaints can signal serious underlying conditions. Timely initiation of high-dose corticosteroids is crucial in the management of ICI-induced myocarditis, as it can rapidly control inflammation and prevent further complications. This case illustrates that conduction disturbances, such as an AV block, may partially or fully resolve after appropriate immunosuppressive therapy. Persistent AV block may occur despite immunosuppressive therapy, underscoring the need for individualized decisions regarding pacemaker implantation based on symptomatology and clinical prognosis. Early detection and intervention are key to mitigating risks and improving outcomes in this rare but serious IR adverse event.
